# Recovery of a Lost Nose

**Published:** 1899-04

**Authors:** 


### Recovery of a Lost Nose.

(Dr. M. K. Kimout, Russia). On December 27th, last year, this patient came into my hospital complaining that she was compelled just before to fight a battle with her better half, who gained the victory by biting off her nose. I dressed the wound antiseptically, after which she was permitted to return home. Three or four hours later the patient appeared again, holding in her hand the missing portion of her nose, which had been found by the police officer. My assistant washed the piece in a solution of sodium chloride, sewed it on by eight very fine sutures, applied antiseptic dressing, and sent the patient home with an order to return to-morrow. On changing the dressing next day, the piece sewed on had a perfectly healthy red color, but on the third day it turned slightly black. On the fourth day a portion of the sutures were taken out; on the fifth day the rest of sutures were removed, and with this the skin peeled off from the piece that had been severed. This skin I have now in my hand. The whole surface of the wound was covered with healthy granulations. On 11th of January, to avoid the deformity of the nose, I transplanted skin by Thiersch's method, taking it from the shoulder, but made a mistake by cutting a shorter piece than was needed to cover the defect, therefore a small surface remained with a scar.

